# Limb Dominance Results from Asymmetries in Predictive and Impedance Control Mechanisms

**DOI:** 10.1371/journal.pone.0093892

**Published:** 2014-04-02

**Authors:** Vivek Yadav, Robert L. Sainburg

**Affiliations:** 1 Department of Neurology, Penn State Milton S. Hershey Medical Center and College of Medicine, Hershey, Pennsylvania, United States of America; 2 Department of Kinesiology, Penn State University, University Park, Pennsylvania, United States of America; University of Reading, United Kingdom

## Abstract

Handedness is a pronounced feature of human motor behavior, yet the underlying neural mechanisms remain unclear. We hypothesize that motor lateralization results from asymmetries in predictive control of task dynamics and in control of limb impedance. To test this hypothesis, we present an experiment with two different force field environments, a field with a predictable magnitude that varies with the square of velocity, and a field with a less predictable magnitude that varies linearly with velocity. These fields were designed to be compatible with controllers that are specialized in predicting limb and task dynamics, and modulating position and velocity dependent impedance, respectively. Because the velocity square field does not change the form of the equations of motion for the reaching arm, we reasoned that a forward dynamic-type controller should perform well in this field, while control of linear damping and stiffness terms should be less effective. In contrast, the unpredictable linear field should be most compatible with impedance control, but incompatible with predictive dynamics control. We measured steady state final position accuracy and 3 trajectory features during exposure to these fields: Mean squared jerk, Straightness, and Movement time. Our results confirmed that each arm made straighter, smoother, and quicker movements in its compatible field. Both arms showed similar final position accuracies, which were achieved using more extensive corrective sub-movements when either arm performed in its incompatible field. Finally, each arm showed limited adaptation to its incompatible field. Analysis of the dependence of trajectory errors on field magnitude suggested that dominant arm adaptation occurred by prediction of the mean field, thus exploiting predictive mechanisms for adaptation to the unpredictable field. Overall, our results support the hypothesis that motor lateralization reflects asymmetries in specific motor control mechanisms associated with predictive control of limb and task dynamics, and modulation of limb impedance.

## Introduction

Substantial differences in control and coordination between the dominant and non-dominant arms are easily recognized by most individuals. Nevertheless, coordination of each arm is seamlessly integrated into activities of daily living, such as donning and doffing clothing, preparing meals, and other common activities. These tasks tend to be performed by distributing action components across the two arms. For example, when slicing meat, the non-dominant hand stabilizes the food with a fork, while the dominant hand manipulates the knife. Previous research has suggested that this distribution of task components to each arm might reflect hemispheric specializations for different aspects of motor control [1] [2] [3] [4] [5] [6]. Based on this idea, we previously elaborated a model of movement lateralization called “dynamic dominance” which attributes predictive control of movement dynamics to the dominant system and the ability to stabilize position in the face of unpredictable or unexpected dynamic conditions to the non-dominant system [7]. We operationalized this model into a computational simulation that accounted for consistent interlimb differences in torque profiles of single joint targeted elbow movements [8]. This simulation predicted differences in the torque profiles of right and left arm movements, without requiring any differences in task performance variables, such as speed or final position accuracy: For the dominant arm, peak acceleration was scaled with target distance while acceleration duration remained fairly constant across targets. For the non-dominant arm, peak acceleration remained fairly constant across targets, while acceleration duration scaled with target distance. Follow-up research in right-handed stroke patients revealed that these differences were reflected in patients with left- and right-hemisphere damage, such that right hemisphere lesions restricted modulation of acceleration duration, whereas, left hemisphere lesions restricted modulation of acceleration amplitude across target distances [9]. These findings supported the proposition that differences in coordination between the arms arise from hemispheric specializations for specific motor control processes.

However, the majority of previous research on motor lateralization has documented asymmetries in performance that are highly task specific, and that overwhelmingly reflect advantages for the dominant arm. These include asymmetries in the timing and/or accuracy in performance of pegboard tasks, writing, drawing, Fitt's tapping tasks, and reaching tasks, among others [10] [11] [12] [13] [14] [15]. The search for advantages for the non-dominant arm has generally resulted in equivocal findings [16] [17]. More recently, studies from our laboratory and others have reported advantages of each arm for different performance measures in the same task, such as reaching or rapid alternating targeted movements [5] [18]. Our research has revealed unambiguous non-dominant arm advantages in the final position accuracy of reaching movements when reaching to a large array of targets without visual feedback [18], or when experiencing unexpected mechanical perturbations during the movements [19]. The mechanistic model of lateralization that we have developed attributes these non-dominant arm advantages to hemispheric specializations for impedance control, which stabilizes performance against unexpected mechanical perturbations and stabilizes final positions, in the face of varied task dynamics. However, this control-based explanation for handedness has remained speculative because dominant arm performance advantages could have emerged from habitual or long-term preferences for specific tasks, such as writing and throwing, or from specializations for perceptual or cognitive aspects of task planning, rather than from motor control processes, per se'. For example, right hemisphere advantages for visual spatial analysis [20] might lead to left arm advantages in final positional accuracy, while more complex features of trajectory planning might be specialized to left-hemisphere processes [21]. Therefore, it is possible that the interlimb differences are manifestation of habitual or long-term preferences for specific tasks, and do not result due to differences in control mechanisms.

*This study was designed to directly test our control-based hypothesis of motor lateralization: that control of the dominant arm is specialized for predictive control of limb and task dynamics, whereas control of the non-dominant arm is specialized for modulation of limb impedance.* A key element of this design is that the *same* performance variables were compared under different dynamic conditions. We designed *two force fields to be compatible with controllers that are 1) specialized in modulating position and velocity dependent impedance or 2) specialized in predicting limb and task dynamics. Both fields were ‘curl’ fields, but one varies with the square of tangential hand velocity while the other varied linearly with tangential hand velocity. Because the velocity square field did not change the form of the equations of motion for the reaching arm, a forward dynamic-type controller should perform well in this field, while control of linear damping and stiffness terms should be less effective. The magnitude of the two fields also differed in predictability: While the velocity square field was consistent in magnitude between trials, the linear field varied in magnitude between trials. This factor should have little impact on an impedance controller, which should be minimally affected by such variability, but should disadvantage a predictive controller, which cannot predict the magnitude of the field from trial to trial. Thus, we designed the fields according to two factors: dynamics (square or linear relation to tangential hand velocity, and predictability (consistent or inconsistent magnitude).*

We employed a 2 Arm (dominant/non-dominant) X 2 field (consistent/inconsistent) design, using four groups of subjects, each of whom performed one session of one load condition with either the right or left arm. We recruited only right-handed subjects because left-handers do not represent a behaviorally- [22] [23] nor a neurologically [24] homogenous population. In addition, our previous studies in stroke patients formed the basis for our primary hypothesis and were only done in right-handers. Right-handedness was assessed using a modified version of the Edinburgh inventory [25].

### Predictions

We hypothesize a dominant system specialization for predictive control of task dynamics, and a non-dominant specialization for impedance control mechanisms. In terms of our dynamic manipulation, this hypothesis predicts that the non-dominant controller should be disadvantaged for performance in the velocity square field, which should require predictive control of limb dynamics. In contrast, the dominant controller should not be disadvantaged in this field, since the field does not change the form of the equations of motion. In terms of our predictability manipulation, the inconsistent field should disadvantage the predictive controller more than the impedance controller. Together, both of these predictions should be supported by a hand (left/right) X field (consistent/inconsistent) interaction for all trajectory measures, such that the dominant arm performs straighter, smoother, and quicker movements in the consistent/predictable, velocity square field, and the non-dominant arm performs straighter, quicker, smoother movements in the inconsistent/unpredictable, linear field. While we expect these predictions to effect performance throughout exposure to the field, we also expect that with repeated experience, the differences in performance between the limbs should become reduced as each arm adapts to each field. In fact, Schabowsky et al. (2007) previously demonstrated that both arms adapt similarly to a consistent velocity dependent curl field, although each employing different mechanisms of control for this adaptation [2].

Our a priori predictions for final position accuracy are less clear because of a number of factors: 1) The imposed fields are velocity dependent and thus become minimized at the final stage of motion 2) Subjects can correct their trajectory errors through submovements in the final phases of motion, and 3) Previous research has indicated that each arm can be advantaged for final position accuracy, under different task parameters [7] [18] [19] [26] [27]. However, we do expect that final position errors should be greater for each arm in its incompatible field, which is the inconsistent linear field for the dominant arm, and the consistent velocity square field for the non-dominant arm.

## Methods

### Subjects

Subjects were recruited from University community and gave written informed consent prior to testing. Twenty four neurologically intact, right-handed adults (18–40 years old) were divided into four groups (3 female and 3 male in each of four groups). The subjects in each group performed the tasks of the experiment with either left or right hand. We used a group design in this study because previous research from our laboratory has shown transfer of learning between the limbs [26].

### Ethics statement

Subjects were recruited from the University community and gave written informed consent prior to testing. All the subjects were paid for their participation. Informed consent and all the experimental procedures were approved by The Pennsylvania State University Institutional Review Board.

### Experimental set-up

**Figure 1:**
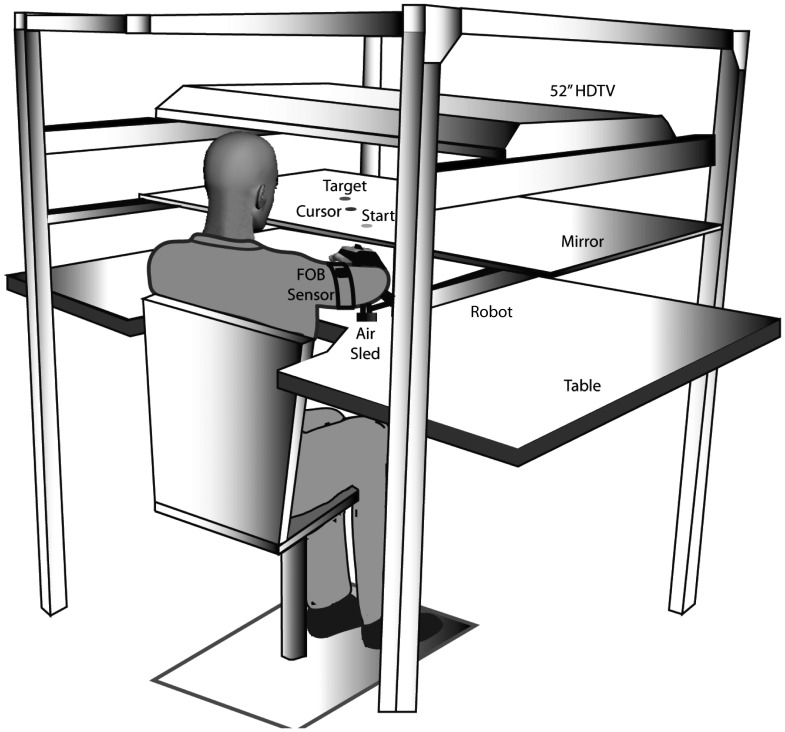
Experimental apparatus.

The experimental set-up is depicted in Figure 1. Subjects sat facing a table with their arm supported in an air-sled over the horizontal surface by an air-jet system. The air-sled was attached to a MIT IMT2 robot arm with an ATI 6-degree of freedom (DOF) force transducer to measure forces at the interface. The robot operated in ‘dynamic inertia compensation mode’ to compensate for the effect of inertia of the robot. The robot arm has back-drivable motors that can be used to apply custom force fields at the hand-robot interface. An LCD screen was positioned above the mirror, which reflected a 2-D virtual reality environment, in which a start position and target were presented. Subjects were instructed to move the hand to a displayed target in response to a “go” signal. Feedback of the fingertip position was given while placing the hand at the start position, but was removed at the “go” signal. Positions and orientations of each segment were sampled at 130 Hz using a Flock of Birds (FOB: Ascension-Technology) electromagnetic 6-degree of freedom movement recording system. Custom computer algorithms for experiment control and data analysis were written in REAL BASIC (REAL Software, Inc.), C and IgorPro (Wavemetric, Inc.).

### Robot Induced Force Fields

The IMT MANUS robot arm has the capability of applying custom force fields, which we specifically designed to test our primary hypothesis of motor lateralization. The equations of motion of human arm interacting with the robot can be written as, 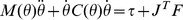
(1)where M is the inertia matrix, C is the tensor of centripetal and Coriolis forces, 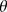 is the vector of joint angles,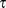 is the torque applied at the joints, F is the force at the interface (force applied by the robot to the hand of the subject) and J is the manipulator Jacobean. If the robot applied force field, F is chosen as a constant times velocity [2], i.e. *F =  −BV*, the equations of motion change as, 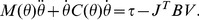
(2)


Our inconsistent force field was defined as,
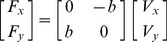
(3)and was simulated by varying *b* between trials. For the right hand, the values changed between 15, 20 and 25. Whereas, for the left hand, *b* values changed between −15, −20 and −25. This ensured that the forces experienced by each hand were symmetric in joint coordinates. Adaptation to such a force field requires generating a velocity dependent force.

However, if the force field is chosen as F =  −*abs*(V^T^)BV, the equations of motion change as
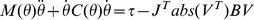
(4)


More specifically, we chose the consistent field as, 
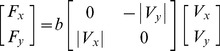
(5)where *b* was 40 for the right hand, and −40 for the left hand movements. This structure of the consistent force field ensured that the applied force field had the same direction as a typical curl field, but its magnitude varied with square of the velocity.

Noting that 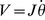
(6)The equations of motion can be rewritten as
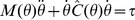
(7)where
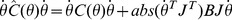
(8)is the modified inertial contributions due to Coriolis and centripetal effects. The structure of Eq. (7) is similar to Eq. (1) with the *C* matrix changed.

#### Choosing force field parameters

By choosing the robot-applied force fields differently, we intended to train either the impedance or predictive neural mechanisms involved in movement generation. However, we also wanted to match the energy requirements of each field, so as not to systematically differ between the groups. In order to estimate the effects of different field strengths (b in Eq. (3) and (5), we assumed a minimum jerk trajectory between initial and final positions [28], and a mean movement time of 450 ms (derived from pilot data). From these, we calculated the average work done at the interface as
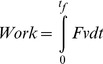
(9)where *F* is force applied by the robot, *v* is the velocity of the hand-robot interface and *t_f_* is the total movement time. Value of *b = 20* for the inconsistent field in Eq. (3), and b = 40 for the consistent field in Eq. (5) gave net work of approximately 1.2 *J/Kg* for both the fields. Therefore, we chose the b-values that characterize individual force fields so that the net work done by a subject at the hand-robot interface following a minimum jerk trajectory to the target is comparable.

### Experiment design

Subjects were seated in the virtual reality set up of Figure 1 and performed reaching movements to three targets starting from a start location so that the involved shoulder and elbow angles were same at start and at each target (start: 25° and 90°; target 1: 40° and 115°; target 2: 50° and 115°; target 3: 60° and 115°, respectively). The average distance between targets and start position was 14 cm.

Subjects performed a total of 207 movements toward the target. The first 27 of these movements were performed under no-load, null field conditions to determine baseline performance for later comparisons. One hundred and eighty movements were performed in the robot-applied force fields. Each trial was initiated by an audiovisual signal at which time the cursor was blanked. Subjects were instructed to move their hand to the target using a single rapid motion, with peak velocity greater than 0.5 m/s. To motivate subjects, audiovisual feedback was provided, and points were given based on accuracy, when peak velocities were greater than this threshold. They were provided graphic feedback indicating whether their movements were too slow, following each trial.

### Statistical and data analysis

Our parametric measures include: 1) Final position error, computed as the Euclidean distance between the position of the hand at the end of reaching movement and the corresponding target. 2) Mean squared jerk, the third derivative of displacement with respect to time, which has previously been exploited to reflect a spatiotemporal measure of general movement quality [29]. 3) Deviation from straight-line performance was measured as the ratio of cumulative hand path length to the straight-line distance between the initial and final point in the path [30] and 4) Movement duration was measured as the time between movement initiation and movement end. Final position error represents the major task performance criterion, while smoothness reflects a combined spatiotemporal measure of performance. Linear deviation and Movement duration provide individual spatial and temporal measures of performance, respectively. We subtracted the performance metrics for baseline (i.e. no field condition) from the performance metrics for the adaptation phases so our comparisons were not affected by potential differences in baseline performance between the groups. We divided data into 10 phases of 18 consecutive trials, which allowed us to investigate the effects of hand and field on adaptation. For analysis, we employed a 2 (arm) X 2 (field) x 3 (target) X 10 (phase) mixed factor ANOVA to test our major hypothesis: That the left (non-dominant) arm should be advantaged for performance in the inconsistent field and disadvantaged for performance in the consistent field. In contrast, the right (dominant) arm should be advantaged for performance in the consistent field and disadvantaged for performance in the inconsistent field. In other words, we predicted a crossed interaction between arm and field. While we expected a main effect of both target and phase, these effects were not associated with our primary hypothesis. However, a three way interaction between arm X field X phase would suggest that the time course of adaptation (change across phase) was effected by our primary manipulations (arm and field). We define the time course of adaptation as the number of phases after which the performance stabilizes or asymptotes. Post hoc comparisons were performed using t-test when warranted.

### Corrective sub-movement analysis

We first determined the duration of the first major submovement, which included the peak tangential hand velocity, and ended at the first minima following that peak [30]. The corrective sub movement duration was taken as the duration of the remainder of the movement, where the end of the movement was determined to be the first minima that fell below 5% of the maximum tangential velocity during the trial. The ratio of the corrective submovement duration to total movement time was our normalized measure of the sub movement phase of motion.

### Inconsistent field performance

Previous work from Scheidt et al (2001) has shown that while moving in a dynamic environment with varying magnitude force fields, subjects used a small number of previous trials to compute the mean force field magnitude, and trajectory errors resulted from field strength variations in each trial about that mean [31]. We expect that the dominant arm may use the same predictive strategy in the current study. In order to test this idea, we performed a repeated measures ANOVA for our trajectory measures, with arm as the between-subject factor, and field strength and phase as within subject factor. If only the dominant arm used a mean predictive strategy, such as that employed in Scheidt et al (2001), then we would predict the dominant arm to be more influenced by changes in the field strength than the non-dominant arm. If, instead, both arms converged on a similar control strategy, then we would not expect differences in how each hand is influenced by field strength. This analysis was only applied to the inconsistent linear field condition, because field strength remained constant in the consistent velocity square field condition.

### Design Confirmation

We tested whether our experimental design might have introduced interlimb differences in force amplitude that could result in performance differences between the arms, which would nullify our experiment. For both of our fields, the forces applied by the robot depended on the magnitude of the velocity. We thus compared maximum velocity between all the groups using a repeated measures ANOVA, with arm and field as the between subject factors, and target and phase as within subject factors. Our ANOVA did not reveal a main effect of arm (F(1,20) = 2.118, P = 0.1611), field (F(1,20) = 0.1710, P = 0.6836) nor an arm by field interaction, (F(1,20) = 0.0273, P = 0.8705). Based on these results, we conclude that peak tangential velocity was not significantly different between the four groups. We next computed the maximum force applied by the robot at the hand-robot interface for a subset of subjects (16 subjects), and performed comparisons using ANOVA. The rationale for this smaller subset of subjects (4 per group) was that we experienced technical problems with our force transducer for the last 2 subjects in each of our groups. ANOVA results showed no significant effect of arm (F(1,12) = 3.074, P = 0.1050, nor field (F(1,12)  = 0.734, P = 0.4084), nor an arm by field interaction (F(1,12)  = 0.181, P = 0.6783). We conclude that any performance differences between the dominant and non-dominant arms did not result from differences in the magnitudes of the imposed fields.

## Results

### Hand paths

**Figure 2:**
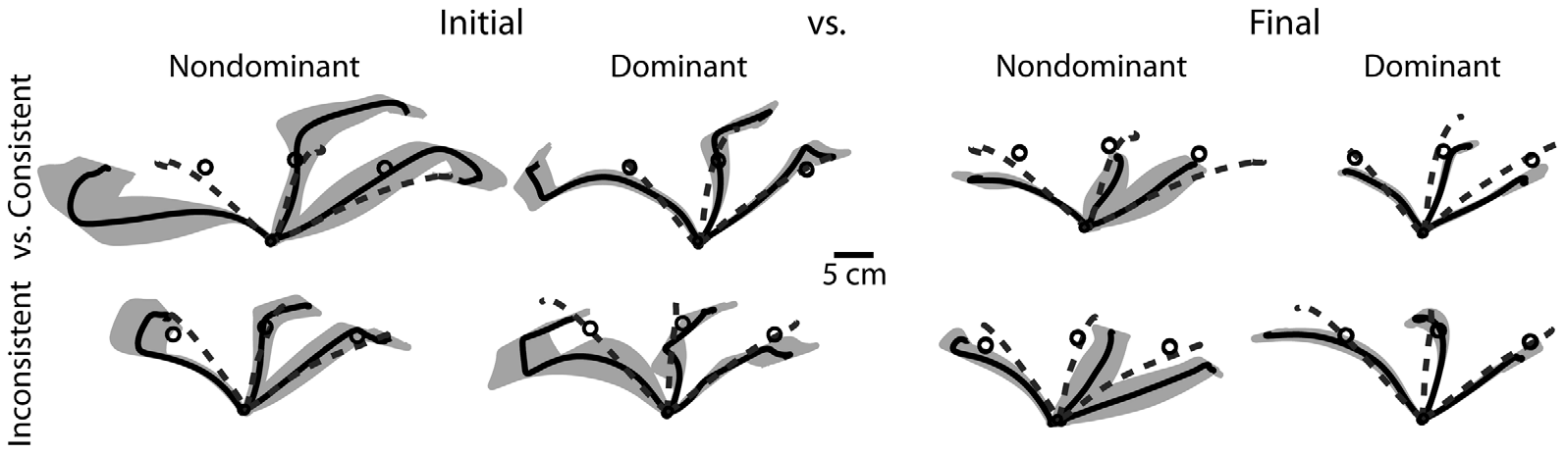
Hand paths in the consistent and inconsistent fields for the dominant and non-dominant arms during initial and final phases of adaptation. The non-dominant arms' hand paths are flipped about the y-axis such that left of the center is medial and the right is lateral for both the dominant and non-dominant arms.

The hand paths in Figure 2 provide a qualitative representation of the general experimental findings. Each path shows the mean of the initial or final phase of adaptation for a representative subject from each condition, with gray representing standard error of the mean. Mean hand paths in the baseline condition are represented by dashed lines. For the initial phase of adaptation, the paths are very curved and final positions inaccurate for both arms in both fields. However, the dominant arm paths are less curved and more accurate in the consistent field, while non-dominant arm paths are less curved and more accurate in the inconsistent field. In the final phase of adaptation, movements become straighter and more accurate for both arms in both fields. In the following sections, we present parametric measures of performance to quantitatively assess these general findings.

### Final position errors

**Figure 3:**
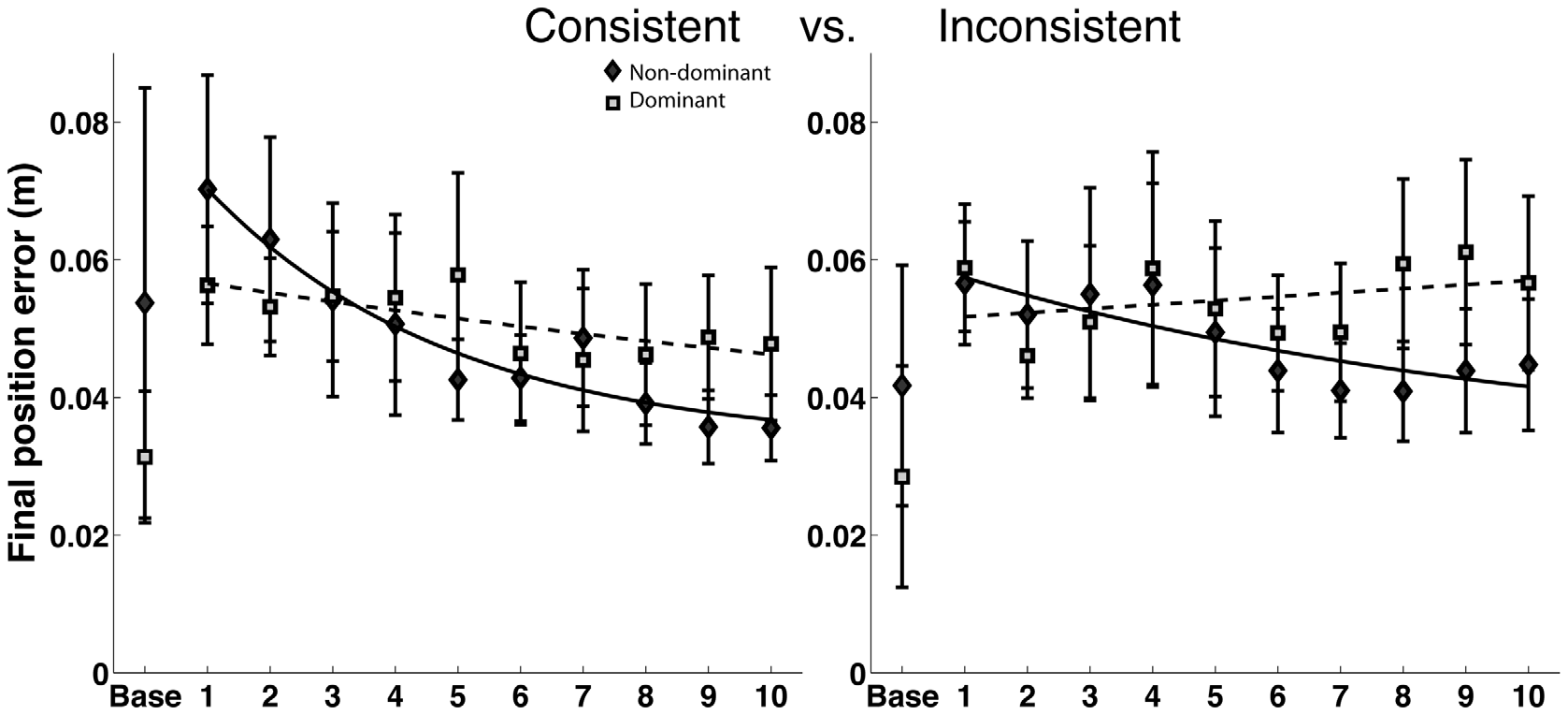
Final position error for the dominant and non-dominant in consistent and inconsistent fields.

Figure 3 shows final position errors for all four groups during all 10 phases of adaptation. The dashed line represents dominant arm performance, while the solid line represents non-dominant arm performance for each field. The most striking feature of these plots is that consistent improvement that the non-dominant arm makes across the 10 phases of adaptation, and the lack of change in final position error for the dominant arm across adaptation. In addition, both arms showed comparable final position errors at the end of adaptation that approximated the diameter of the target (5 centimeters). Because of slight differences in performance in the baseline condition (null field), we subtracted null field performance from exposure performance for statistical analysis. Our ANOVA did not show a main effect of arm (F(1,20) = 1.183, P = 0.2897), nor arm by field interaction (F(1,20) = 0.0775, P = 0.7836). However, the tendency for the non-dominant arm, but not the dominant arm, to improve over adaptation was reflected in a significant arm by phase interaction (F(9,580) = 2.003, P = 0.0368*). Post-hoc comparisons confirmed that non-dominant arm errors in the final phase of adaptation were significantly lower than in the initial phase (P<0.0001*), whereas dominant arm errors were not significantly different between initial and final phases of adaptation (P = 0.3410). Previous studies have shown greater improvements in accuracy for the non-dominant arm during adaptation to novel dynamic environments [1] [2], and had attributed these results to a non-dominant arm advantages in control of steady state position, although the trend for the non-dominant arm to show greater accuracy by the end of adaptation was not significant. It should be stressed that because our applied fields were velocity dependent, they were near-zero in magnitude at the final position, when velocities were near zero.

### Corrections

**Figure 4:**
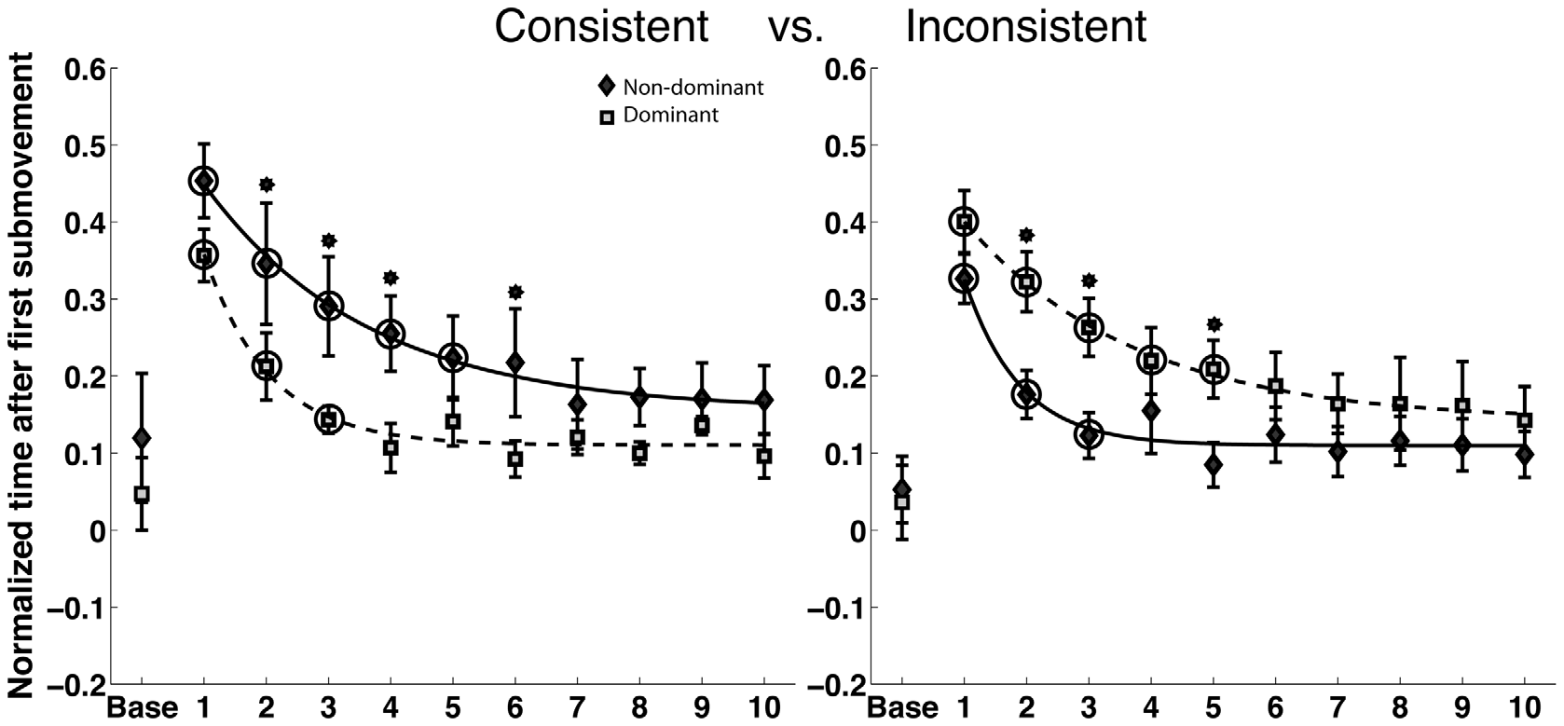
Normalized time after first submovement for the dominant and non-dominant arms in the consistent and inconsistent fields. The asterisks on top of standard error bars indicate the phases where the interlimb differences were significant in post-hoc tests. The circles indicate the phases beyond which there was no significant difference between the final phase and individual phases.

The hand-paths in Figure 2 left columns show that when initially exposed to the force fields, subjects' hand paths are substantially misdirected and curved. However, they also show that substantial corrective sub movements were made at the end of motion, which often directed the hand back toward the target. We expected that the equivalent final position errors made with both hands in both fields, resulted from such corrections at the end of motion. To assess this, we measured the duration of the corrective phase, following the initial movement (see methods). Figure 4 presents the ratio of this duration to total movement duration. While the final positions of the two arms were the same in both fields, this analysis reveals that the dominant arm had a shorter corrective phase than the non-dominant arm in the consistent velocity square field, while the non dominant arm had a shorter corrective phase in the inconsistent linear field. This trend was reflected by a significant hand by field interaction (F(1,20)  = 6.21, P = 0.0216*). A significant 3-way interaction between phase, hand and field (F(9,580)  = 4.0836, P = 0.0240*) revealed that the time course of adaptation was also different. The non-dominant arm showed faster reductions in the corrective durations than the dominant arm in the inconsistent field. However, the dominant arm showed faster reductions in corrective durations than the non-dominant arm in the consistent field. Thus, both arms achieved equivalent final position accuracies across fields by exploiting greater corrective processes when performing in its incompatible field.

### Mean squared jerk (spatiotemporal measure)

**Figure 5:**
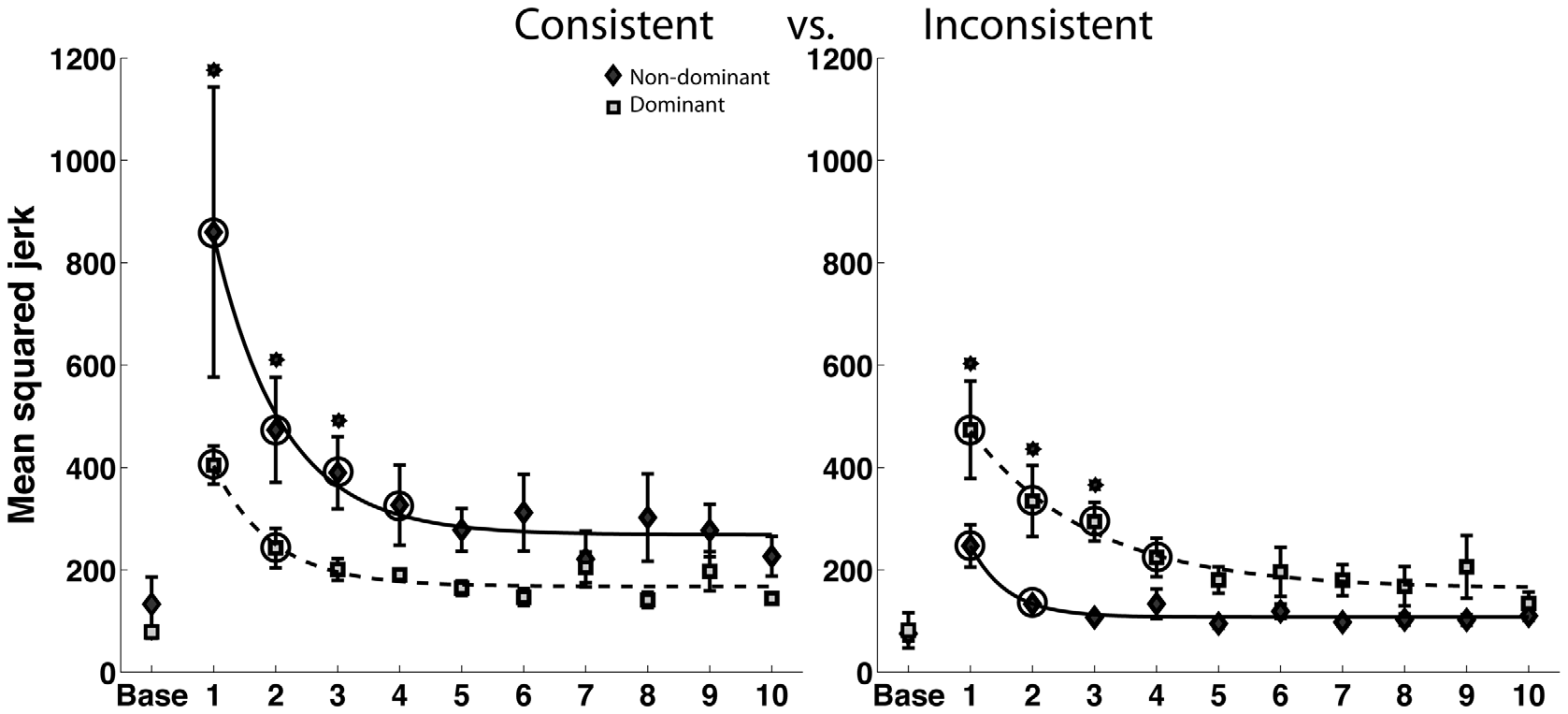
Mean squared jerk for the dominant and non-dominant arms in the consistent and inconsistent fields. The asterisks on top of standard error bars indicate the phases where the interlimb differences were significant in post-hoc tests. The circles indicate the phases beyond which there was no significant difference between the final phase and individual phases.

We quantified interlimb differences in mean squared jerk throughout the course of adaptation, as a general indication of movement quality (Figure 5). Our ANOVA revealed a significant main effect for phase, reflecting reductions in jerk for both arms under both fields (F(9,580) = 21.37,P<0.0001*). A significant hand by field interaction (F(1,20) = 10.72, P = 0.0038*) occurred, reflecting lower jerk for the dominant arm in the consistent field, and for the non-dominant arm in the inconsistent field. A three way interaction between phase, arm and field (F(9,580) = 4.06, P<0.0001*) also suggested that the time course of adaptation for the two arms in the consistent and inconsistent fields were different. To investigate this further, we performed post hoc comparisons of mean squared jerk between the final phase and previous phases for each arm in each field. The cycles after which there was no significant difference between individual phases and the final phase, are marked with circles in Figure 5. These results suggest that non-dominant arm performance stabilized earlier than dominant arm performance in the inconsistent field, and dominant arm performance stabilized earlier in the consistent field. Furthermore, this 3-way interaction suggests that the interlimb differences between the arms for the two fields varied differently across the phases of adaptation. In order to examine this effect in more detail, we performed post-hoc analysis for each phase of the session. The phases of adaptation for which the interlimb differences were significant are marked by asterisks in Figure 5. Our post hoc comparisons revealed that interlimb differences in jerk were resolved in both fields by the fourth phase of adaptation, and that this convergence was sustained throughout the session. These results reveal a symmetry in convergence for our combined spatio-temporal measure, such that in both the fields, the dominant and non-dominant arms converged to a similar extent by the end of adaptation.

### Linearity Deviation (spatial measure)

**Figure 6:**
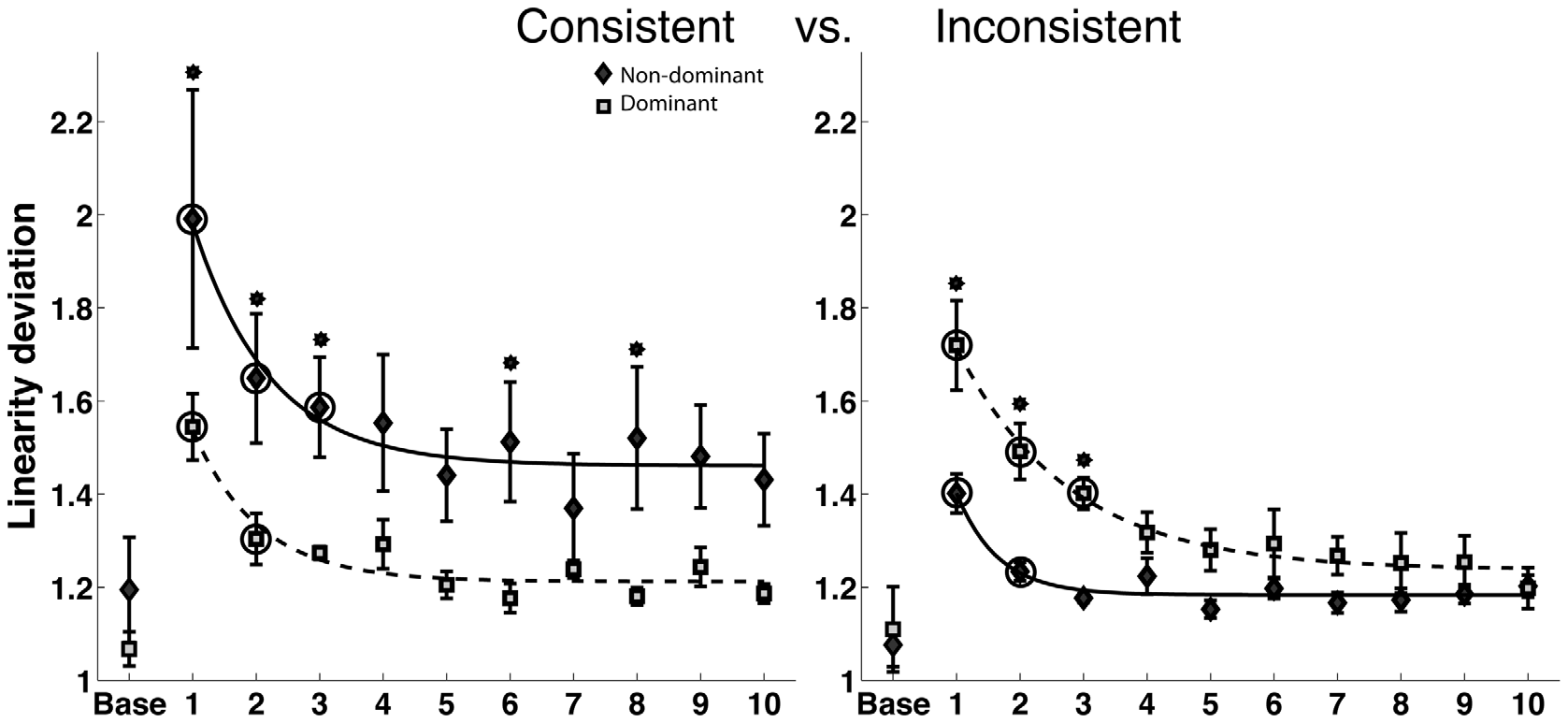
Linearity deviation for the dominant and non-dominant arms in the Consistent and Inconsistent fields. The asterisks on top of standard error bars indicate the phases where the interlimb differences were significant in post-hoc tests. The circles indicate the phases beyond which there was no significant difference between the final phase and individual phases.

We next assessed hand-path deviation from linearity, as a purely spatial measure of the hand-path during the trajectory phase of motion. Figure 6 compares mean (+/− standard error) profiles across adaptation, for both arms in each field. When exposed to the fields, both arms showed substantial improvements through adaptation, as reflected by a main effect of phase in our ANOVA (F(9,580) = 17.76, P<0.0001*). However, the dominant arm showed substantially straighter movements in the consistent field, whereas the non-dominant arm showed straighter movements in the inconsistent field, an effect quantified as a significant arm X field interaction (F(1,20) = 6.59, P = 0.0184*). A three way interaction between arm, phase and field (F(9,580)  = 2.04, P = 0.0331*) revealed that the time course of adaptation was different for the two arms in the two fields. The dominant arm adapted in fewer phases than the non-dominant arm in the consistent field, where as the non-dominant arm adapted in fewer phases in the inconsistent field. An asymmetry in adaptation was also reflected by a significant 3-way Arm X Field X Phase interaction in our ANOVA. During the course of adaptation, the straightness of dominant arm movements converged toward that of non-dominant arm movements by the last phase of adaptation. However, non-dominant arm movement straightness did not converge to dominant arm levels under the consistent field. As expected, in the initial phase of consistent field adaptation, dominant hand paths were substantially straighter than non-dominant paths (P = 0.0015*), whereas, in the initial phase of inconsistent field adaptation, non-dominant paths were straighter (P = 0.0060*). For the inconsistent field, dominant arm paths showed significantly greater linearity deviation for the first three phases, but converged to that of non-dominant arm performance by the fourth phase. However, in the consistent field, non-dominant arm movements remained less straight than dominant arm movements as late as the 8th phase. Although, not all phases prior to this were significantly different, slight convergence suggested by phases 4 and 5 were not sustained. We thus conclude that the crossed interaction between hand and field for linearity was strongest early in adaptation, but convergence in this measure depended on the field.

### Movement Duration (temporal measure)

**Figure 7:**
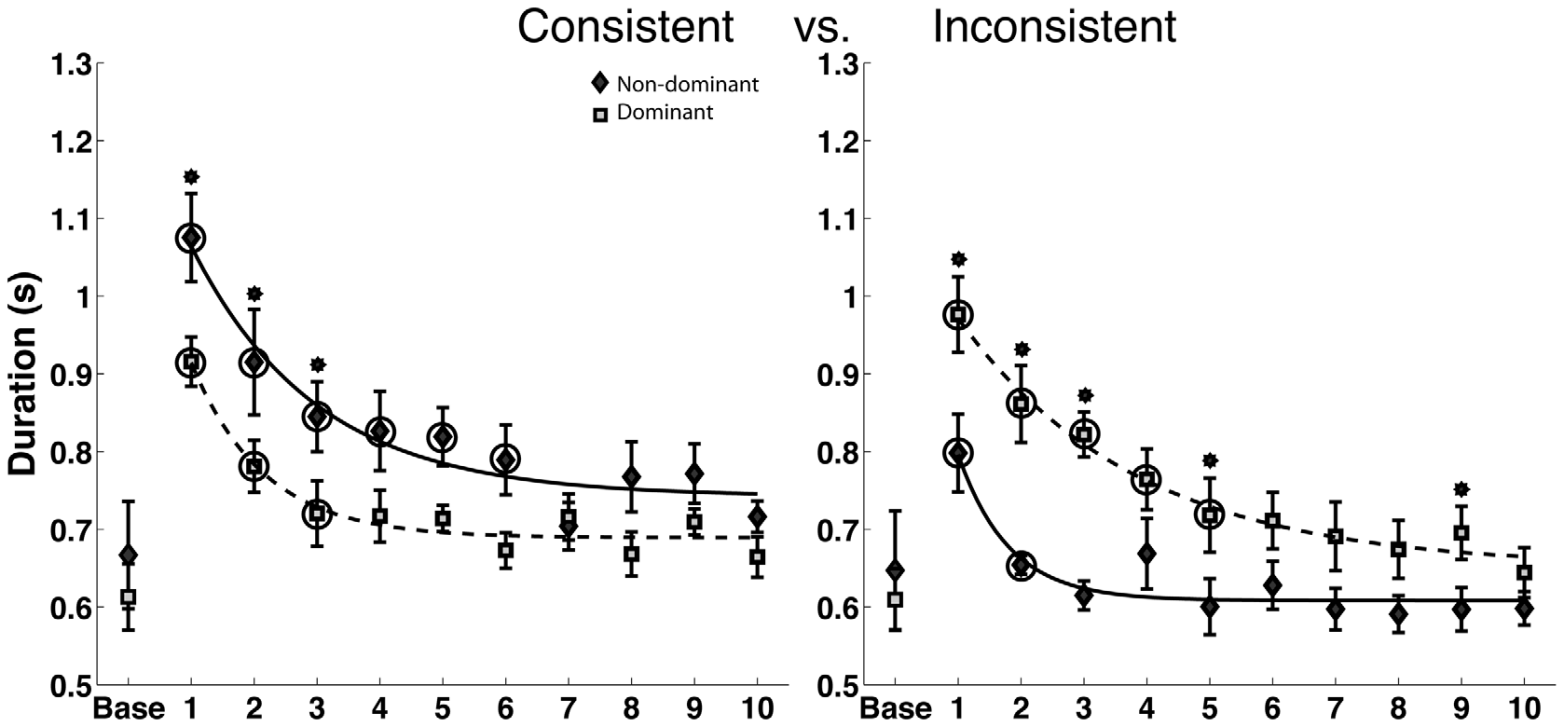
Duration for the dominant and non-dominant arms in the consistent and inconsistent fields. The asterisks on top of standard error bars indicate the phases where the interlimb differences were significant in post-hoc tests. The circles indicate the phases beyond which there was no significant difference between the final phase and individual phases.

Figure 7 shows movement durations, quantified for each group, across the phases of adaptation in both fields. Our ANOVA revealed a significant interaction between hand and field (F(1,20) = 10.85,P = 0.0036*), a significant main effect of phase (F(9,580) = 27.59,P<0.0001*), and a significant 3-way interaction between arm, field and phase (F(9,580) = 2.16,P<0.0230*). A 3-way interaction between arm, field and phase revealed a difference in time course of adaptation for the two arms such that the dominant arm's performance stabilized earlier than the non-dominant arm in the consistent field, and the non-dominant arm stabilized earlier in the inconsistent field. Furthermore, our post hoc analysis of phase (indicated by asterisks) indicated that for the consistent field, movement duration converged between the hands by the fourth phase of adaptation, and that this convergence was sustained. In contrast, in the inconsistent field, dominant arm duration was significantly higher than non-dominant arm duration as late as the 9th phase of adaptation.

### Influence of field strength on dominant and non-dominant arm performance in the inconsistent field

**Figure 8:**
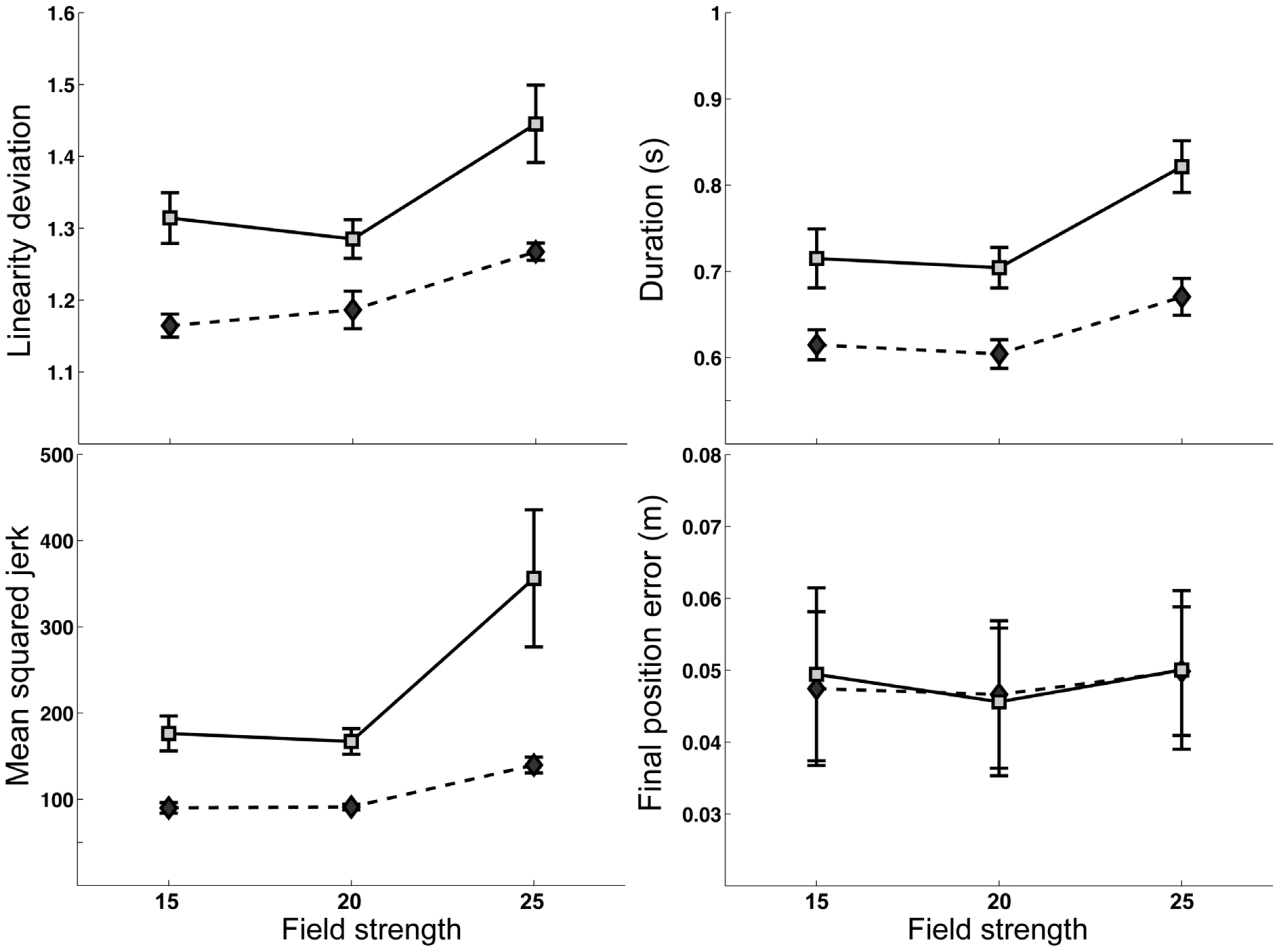
Performance measures for the dominant (shaded-square) and non-dominant (dark-diamond) arms in unpredictable field for different field strengths.

We further explored the plausible mechanism underlying adaptation to the inconsistent field. In this field, the field strength varied across 3 gains, in a randomized trial sequence, such that the strength was not predictable in any trial. However, Scheidt et al,(2001) previous studied how the dominant arm adapts to such unpredictable situations [31]. When subjects are exposed to curl fields that vary in strength between trials, they tend to develop a predictive strategy, using the mean strength of a previous few trials. Trajectory errors depend upon the difference in the current force field strength from this predicted mean. Thus, it is possible that in the current study, subjects may have employed a similar strategy. We tested this hypothesis by subjecting our performance measures to a repeated measures ANOVA with field strength and phase as within-subject factor, and arm as between subject factor. Our ANOVA revealed a significant interaction between arm and field-strength for linearity deviation (F(2,290)  = 3.724, P = 0.0253*), mean squared jerk (F(2,290)  = 10.132, P<0.001*) and movement duration (F(2,290)  = 3.403, P = 0.0346*). Final position error did not show a significant interaction between field strength and arm (F(2,290)  = 0.357, P = 0.6999). Figure 8 presents final position error and our 3 trajectory measures for the dominant and non-dominant arms in the inconsistent field. The dominant arm's performance was more influenced by the magnitude of the field, especially for high field strength conditions. Interestingly, the dominant arm also showed a minimum in errors in the middle magnitude condition, corresponding to the mean field magnitude. While this trend occurred for the dominant arm for all measures, performance in the mean field conditions was not significantly different than the low magnitude field condition (Table 1). In contrast, the non-dominant arm's trajectory errors showed lower dependence on field, and the middle amplitude field tended to be the same or higher than the low magnitude field condition. Thus, dominant arm performance was consistent with a predictive mean-field strategy, whereas, non-dominant arm performance was not.

**Table 1:** Post-hoc comparisons between fields of different magnitudes.

Metric/Comparison	15 vs 20	20 vs 25	15 vs 25
Movement time	(0.508, 0.500)	(<0.001*,<0.001*)	(<0.001*,<0.001*)
Linearity deviation	(0.294, 0.161)	(<0.001*,<0.001*)	(<0.001*,<0.001*)
Mean integrated jerk	(1.000, 0.999)	(0.357, <0.0001*)	(0.332, <0.0001*)
Final position error	(0.747, 0.132)	(0.204, 0.081)	(0.342, 0.812)

P-values from comparisons are presented for left and right hands (Left, Right). Statistically significant differences are marked by asterisks.

## Discussion

Handedness is a prominent and ubiquitous feature of human motor behavior, yet the underlying causative factors that produce the associated movement asymmetries remain incompletely understood. This study was designed to test a dual-control hypotheses of motor lateralization: Motor lateralization results from underlying asymmetries in control mechanisms, including predictive control of task dynamics, and control of limb impedance. In order to test this hypothesis, we presented a group design with two different force field environments, an inconsistent velocity dependent curl field, and a consistent velocity-square dependent curl field. We designed the two fields so that the net work for straight line movement was similar between the fields. As the hand paths were not straight line paths in the initial phases of adaptation, we used ANOVA to confirm that the forces applied by the robot were similar between the four hand by field conditions. Both, our force field design and empirical data revealed no field by hand interaction for the applied force fields. Therefore, we conclude that the observed differences in our dependent measures did not arise due to differences in the magnitudes of force fields, and were due to differences in each arm's control specialization. Our dependent variables included final position accuracy and 3 trajectory measures: Mean squared jerk, Straightness, and Movement time. Our hypothesis predicted a crossed interaction between hand X field for our trajectory measures: The non-dominant arm should demonstrate smoother, straighter, and quicker movements in the inconsistent field, as compared with the dominant arm. In contrast, the dominant arm should show straighter, smoother and quicker performance in the consistent field. Our results support the hypothesis that the two arm controllers are specialized for distinct motor control processes, akin to predictive and impedance mechanisms. As expected, we observed interlimb differences in the initial phase of adaptation, while these differences were reduced as each arm progressively adapted to each field. Previous work comparing interlimb differences in adaptations to a fixed curl field revealed that the differences in the dominant and non-dominant arms were substantial in the first few trials of movement [2]. We expect that these differences result from the differences in control strategy employed, even though subjects have little experience with the applied force fields. It should be stressed that different control strategies should not result in the same errors, when exposed to a novel field. In fact, our analysis of the dependence of error magnitude on field strength in the inconsistent field demonstrated a greater dependence of the dominant arm on field strength than the non-dominant arm. We interpret this finding as indicative of a predictive strategy that assumes a stable field strength. Early in exposure, this strength is likely to be reflected by the null field, while later, it is clearly supplanted by the mean field. In contrast, non dominant arm errors were less dependent on field strength, consistent with a strategy that is less dependent on field amplitude predictions, such as an impedance control strategy.

Although, our trajectory measures showed a clear arm by field interaction, final position accuracy did not, and was equivalent across both arms and fields. While this finding appears at odds with some previous findings from our laboratory [8], [18], [19], we have reported equivalent final position accuracies for both arms under some conditions, including adaptation to novel environments [7] [26]. For example, we have previously reported similar final position accuracies for rapid single joint movements, even though the movements were associated with different movement control strategies [8]. In another study, we showed a non-dominant arm advantage, when such movements were unexpectedly perturbed by an inertial load. However, in that study we required subjects to reach with a single quick motion, and not to make corrective submovements. We attributed the non-dominant arm advantage to the use of a positional impedance control strategy that simply tracked the arm into the previously specified final position. In the current study, the substantial trajectory errors that occurred required substantial corrective submovements. It should be stressed that in this study, the trajectory errors induced by the two fields were different for the two arms, and we allowed corrective submovements, after the movement. These conditions allowed both arms to achieve equivalent accuracies. We assume that the corrections might have employed different strategies with equivalent results, as previously reported [30]. In support to this interpretation, the accuracy of the non-dominant arm improved throughout practice in both fields, while the accuracy of the dominant arm did not. This might be attributable to an improved impedance control strategy that specified positional impedance at the end-point regardless of error magnitude.

Our results indicate that each arm was able to adapt, to some extent, when performing in its incompatible field. We were able to examine the mechanisms of this adaptation in the inconsistent field by assessing the dependence of error magnitude on field strength: Our results indicated that the dominant arm was more influenced by changes in field strength, than the non-dominant arm. This suggests that the dominant arm employed a predictive strategy, previously reported by Scheidt et al (2001), which was adapted to the mean of the fields [31]. The dominant arm showed a trend toward slightly higher errors than the mid-strength field, supporting this interpretation. This pattern did not occur for the non-dominant arm, which showed a simple, and lower dependence on field strength. While we could not perform a similar analysis for the consistent field, previous research has indicated that the non-dominant arm adapts to a consistent velocity dependent curl field through modulation of limb impedance, while the dominant arm adapts through predictive mechanisms [2]. We interpret these results as indicating that each field modulated its unique control mechanism for adaptation.

### Motor lateralization is based on asymmetries in control-mechanisms

Our major findings revealed a crossed interaction between field and arm for our trajectory measures. This was expressed as dominant arm performance that was straighter, quicker, and smoother in the consistent-velocity square dependent field, yet more curved, slower, and less-smooth in the inconsistent velocity-dependent field. In contrast, the non-dominant arm produced straighter, quicker, and smoother trajectories in the inconsistent velocity dependent field, and more curved, slower, and less-smooth trajectories in the consistent velocity square dependent field. This crossed interaction indicates that control of each arm benefited from the field that was designed for the hypothesized specialization of its controller: Predictive control of task dynamics for the dominant arm and impedance-like control for the non-dominant arm. The importance of this finding is emphasized by the fact that each arm demonstrated ‘dominant’ performance in a one particular field, and ‘non-dominant’ performance in the alternate field. This double dissociation between arm and field is strong evidence that each arm has become specialized for different processes and against the propositions that handedness reflects an overall advantage for the dominant arm. We propose that our current findings represent a substantial advance in understanding the mechanisms that underlie motor lateralization in humans.

### Predictive and Impedance Control Mechanisms

The idea that motor control mechanisms can be represented by a hybrid of predictive and impedance-like control has previously been supported and computationally operationalized [32] [33] [34] [35]. In addition, these ideas have more recently gained support from empirical studies [36] [37] [38] [39] [40]. Predictive control mechanisms seek to optimize a combination of kinematic and dynamic costs of movement [8] [29] [36]. Examples of component costs that have been proposed in the literature include Movement Smoothness, Mean Squared Torque, Peak Work, Muscle Energy and Final Position Variability [29] [41] [42] [43] [44]. Many studies have suggested that such costs may be considered during movement planning, even though subjects do not have declarative knowledge of these considerations. However, predictive control based on such optimization principles, whether implemented through open loop or optimal feedback control schemes [45], is not robust to sudden and substantial changes in task dynamics or unanticipated perturbations. As a result, predictive mechanisms can be cost-efficient, but are not well-suited to conditions of environmental instability or uncertainty. In contrast, impedance control mechanisms are well-suited to such unpredictable conditions. Mechanical impedance refers to “resistance to movement”, and impedance control is achieved by changing the effective stiffness-like and viscous-like behavior of the limb [46]. This can be achieved by a combination of muscle co-activation [37] [47] [48], and modulation of reflexes [49] [50]. In fact, some studies have suggested that impedance mechanisms provide stability during the initial phases of motor learning, when predictive mechanisms are not effective in error prevention [31] [51] [52], or when environmental conditions are too unstable to be predicted [51] [53]. For example, Wei et al (2010), showed that when subjects are exposed to a random sequence of novel visuomotor and mechanical fields (ramp, half-sine, sine, double sine, and triangle), the subject's performance on a given trial does not depend on the nature of the perturbation on the previous trial, supporting a role for impedance mechanisms under such conditions [53]. Recent findings during adaptation to novel force environments have suggested that the non-dominant arm tends to rely on impedance control for adaptation, even when conditions are predictable, whereas, the dominant arm tends to rely on predictive mechanisms [1] [2] [26]. A major limitation of impedance control mechanisms, however, is that they cannot be used to optimize factors such as energy expenditure, and thus can result in high energetic costs. This is consistent with the finding that the non-dominant arm, which relies on such control, tends to perform movements with higher energetic cost than the dominant arm [7] [54] [55]. Thus, each control scheme offers advantages, which can counter the disadvantages of the alternate control scheme. Our current findings support the idea that these two mechanisms have become differentially specialized to each arm system. Although, previous studies in stroke patients suggest that the two hemispheres have become specialized for these different control strategies [56] [57] [58] [59] [60] [61] [62], we cannot make a direct association between hemisphere and arm performance in the current study.

### Hybrid Control Mechanisms

The current study directly tested our hypothesis of differential control mechanisms for the dominant and non-dominant hemisphere/arm systems [7] [63]. We previously operationalized this hypothesis into a computational model, which incorporated a predictive controller, based on optimization of task and energetic costs, and an impedance controller with separate position and velocity dependent gains [8]. In this simulation, the two controllers were combined in series, such that all movements were initiated by the predictive controller, and terminated by the impedance controller. We fit this simulation to dominant and non-dominant single joint elbow movements recorded from subjects, by leaving open the time to switch from the predictive to the impedance controller, as well as the control gains. Our results indicated that when optimally fit to data, non-dominant arm movements were characterized by early switches to impedance control that acted to drive the movements to peak velocity, while dominant arm movements relied on predictive control throughout most of the trial, switching to impedance control to stabilize the final position. Previous research from our laboratory has suggested that these two aspects of control have become specialized in different hemisphere/limb systems: The right hemisphere/limb system for controlling impedance, and the left for predicting task dynamics [63]. For example, we previously showed that stroke patients with lesions of the left cortical lesions show deficits in trajectory performance, including making straight and energetically efficient targeted movements [58] [59] [62]. However, patients with matched right cortical lesions show deficits in final position accuracy and stabilization. While we recently demonstrated that these deficits occur in both the contralesional arm [61] [64], our previous studies demonstrated that the ipsilesional arm also shows these consistent patterns of deficits. These findings suggest that ipsilesional cortex must contribute its specialized motor control processes to both the ipsilateral and contralateral arm. It is plausible that in the current study, adaptation of each arm in its incompatible field may have, at least partially, occurred through recruitment of the other arm's control scheme: impedance control for the dominant arm and predictive control for the non-dominant arm. However, our analysis of dependence of trajectory errors on field strength suggests that, in the current study, the dominant arm relied heavily on its primary controller for adaptation to its incompatible field.

## Conclusions

We propose that the central nervous system invokes two main mechanisms of control to achieve coordinated movements. First, predictive control reflects optimal coordination patterns that satisfy both costs associated with task performance and energetic costs. Second, the nervous system appears to set control policies that modulate sensorimotor circuits such as reflexes, to account for perturbations from unexpected changes in environmental and internal conditions. The current study was designed to directly test the hypothesis that these two strategies reflect specializations that underlie motor lateralization. Our findings support the idea that control of each arm has become specialized for a different type of control: Impedance control for the non-dominant arm, and predictive control for the dominant arm. These findings allow the extension of our understanding of motor lateralization to specific control mechanisms. However, our findings also indicated that with practice, each arm was able to adapt to the field for which it initially showed substantial disadvantages in control. Based on previous research in unilaterally lesioned stroke patients, we speculate that this adaptation may have occurred through recruitment of ipsilateral control scheme. However, our findings indicate that dominant arm adaptation to the inconsistent force field occurred, at least to a large extent, through predictive mechanisms, based on the mean of the field strengths experienced. Further research is necessary to determine a plausible association between the current findings and cortical mechanisms. Specifically, whether right hemisphere lesions prevent adaptation to inconsistent fields, and whether left hemisphere lesions prevent adaptation to consistent fields. These predictions directly stem from a control mechanism based model of motor lateralization. It is important to emphasize that our current findings reveal that the term “dominance” must be used relative to the salient environmental conditions. Under inconsistent dynamic conditions, the traditionally non-dominant arm exhibits dominance, whereas under consistent dynamic conditions, the opposite is true. Thus arm dominance should be considered in relative, rather than absolute, terms.

## References

[1] Duff SV, Sainburg RL (2007) Lateralization of motor adaptation reveals independence in control of trajectory and steady-state position. Exp Brain Res. 179(4): 0551–561 10.1007/s00221-006-0811-1 PMC10681153 17171336

[2] Schabowsky CN, Hidler JM, Lum PS (2007) Greater reliance on impedance control in the non-dominant arm compared with the dominant arm when adapting to a novel dynamic environment. Exp Brain Res 182(4): 0567–577 10.1007/s00221-007-1017-x 17611744

[3] Haaland KY (2007) Hemispheric Dominance for Different Aspects of Movement. Motor Control. 11(Supplement): S7–S8.

[4] Haaland KY, Elsinger CL, Mayer AR, Durgerian S, Rao SM (2004) Motor sequence complexity and performing hand produce differential patterns of hemispheric lateralization. J Cogn Neurosci. 16(4): 621–636 10.1162/089892904323057344 15165352

[5] Winstein CJ, Pohl PS (1995) Effects of unilateral brain damage on the control of goal-directed hand movements. Exp Brain Res. 105(1): 163–174 10.1007/BF00242191 7589312

[6] Sainburg RL (2010) Lateralization of Goal-Directed Movement. In: Elliott D, Khan M, editors. Vision and Goal-Directed Movement: Neurobehavioral Perspectives. Champaign, IL: Human Kinetics. 219–238.

[7] Sainburg RL (2002) Evidence for a dynamic-dominance hypothesis of handedness. Exp Brain Res. 142(2): 241–258 10.1007/s00221-001-0913-8 PMC10710695 11807578

[8] Yadav V, Sainburg RL (2011) Motor lateralization is characterized by a serial hybrid control scheme. Neuroscience. 196: 153–167 10.1016/j.neuroscience.2011.08.039 PMC3199140 21889579

[9] Schaefer SY, Haaland KY, Sainburg RL (2007) Ipsilesional motor deficits following stroke reflect hemispheric specializations for movement control. Brain. 130(8): 2146–2158 10.1093/brain/awm145 PMC3769213 17626039

[10] Annett J, Annett M, Hudson PTW, Turner A (1979) The control of movement in the preferred and non-preferred hands. Qtr J Exp Psychol. 31: 641–652 10.1080/14640747908400755 534286

[11] Boulinguez P, Nougier V, Velay JL (2001) Manual asymmetries in reaching movement control. I: Study of right-handers. Cortex. 37(1): 101–122 10.1016/S0010-9452(08)70561-6 11292156

[12] Todor JI, Kyprie PM (1980) Hand differences in the rate and variability of rapid tapping. J Motor Behav. 12(1): 57–62. 10.1080/00222895.1980.10735205 15215068

[13] Roy EA, Elliott D (1986) Manual asymmetries in visually directed aiming. Can. J Psychol. 40(2): 109–121 10.1037/h0080087 3730950

[14] Todor JI, Cisneros J (1985) Accommodation to increased accuracy demands by the right and left hands. J Motor Behav. 17(3): 355–372. 10.1080/00222895.1985.10735354 15140687

[15] Roy EA, Elliott D (1989) Manual asymmetries in aimed movements. Qtr J Exp Psychol. 41(3): 501–516 10.1080/14640748908402379

[16] Carson RG, Chua R, Elliott D, Goodman D (1990) The contribution of vision to asymmetries in manual aiming. Neuropsychologia. 28(11): 1215–1220 10.1016/0028-3932(90)90056-T 2290495

[17] Elliott D, Roy EA, Goodman D, Carson RG, Chua R, et al (1993) Asymmetries in the preparation and control of manual aiming movements. Can J Exp Psychol. 47(3): 570–589 10.1037/h0078856

[18] Przybyla A, Coehlo CJ, Akpinar S, Kirazci S, Sainburg RL (2013) Sensorimotor performance asymmetries predict hand selection. Neuroscience. 228: 349–360 10.1016/j.neuroscience.2012.10.046 PMC3714798 23111126

[19] Bagesteiro LB, Sainburg RL (2003) Non-dominant arm advantages in load compensation during rapid elbow joint movements. J Neurophysiol. 90(3): 1503–1513 10.1152/jn.00189.2003 PMC10704424 12736237

[20] Hellige JB (1996) Hemispheric asymmetry for visual information processing. Acta Neurobiologiae Experimentalis. 56(1): 485–497. 10.55782/ane-1996-1151 8787209

[21] Winstein CJ, Grafton ST, Pohl PS (1997) Motor task difficulty and brain activity: investigation of goal-directed reciprocal aiming using positron emission tomography. J Neurophysiol. 77(3): 1581–1594. 10.1152/jn.1997.77.3.1581 9084621

[22] Bryden MP (1977) Measuring handedness with questionnaires. Neuropsychologia. 15(4–5): 617–624 10.1016/0028-3932(77)90067-7 896019

[23] Borod JC, Caron HS, Koff E (1984) Left-handers and right-handers compared on performance and preference measures of lateral dominance. Br J Psychol 75(2): 177–186 10.1111/j.2044-8295.1984.tb01889.x 6733391

[24] Kim SG, Ashe J, Hendrich K, Ellermann JM, Merkle H, et al (1993) Functional magnetic resonance imaging of motor cortex: hemispheric asymmetry and handedness. Science. 261(5121): 615–617 10.1126/science.8342027 8342027

[25] Oldfield RC (1971) The assessment and analysis of handedness: the Edinburgh Inventory. Neuropsychologia. 9: 97–113 10.1016/0028-3932(71)90067-4 5146491

[26] Wang J, Sainburg RL (2004) Interlimb transfer of novel inertial dynamics is asymmetrical. Journal of Neurophysiology. 92(1): 349–360 10.1152/jn.00960.2003 PMC10709821 15028745

[27] Wang (2008) A dissociation between visual and motor workspace inhibits generalization of visuomotor adaptation across the limbs. Exp Brain Res, 187: , 2790–2799 doi: 10.1007/s00221-008-1393-x. 10.1007/s00221-008-1393-x PMC2398649 18437367

[28] Flash T, Hogan N (1985) The coordination of arm movements: an experimentally confirmed mathematical model. J Neurosci. 5(7): 1688–1703. 10.1523/JNEUROSCI.05-07-01688.1985 PMC6565116 4020415

[29] Hogan N, Sternad D (2009) Sensitivity of smoothness measures to movement duration, amplitude, and arrests. J Mot Behav. 41(6): 529–534 10.3200/35-09-004-RC PMC3470860 19892658

[30] Shabbott BA, Sainburg RL (2008) Differentiating between two models of motor lateralization. J Neurophysiol. 100(2): 565–575 10.1152/jn.90349.2008 PMC2525729 18497366

[31] Scheidt RA, Dingwell JB, Mussa-Ivaldi FA (2001) Learning to move amid uncertainty. J Neurophysiol. 86(2): 971–985. 10.1152/jn.2001.86.2.971 11495965

[32] Gottlieb GL (1993) A Computational Model of the Simplest Motor Program. J Mot.Behav. 25(3): 153–161 10.1080/00222895.1993.9942046 12581986

[33] Gottlieb GL (1996) Muscle compliance: implications for the control of movement. Exercise & Sport Sciences Reviews. 24: 1–34 10.1249/00003677-199600240-00003 8744245

[34] Gottlieb GL (1998) Muscle activation patterns during two types of voluntary single-joint movement. J Neurophysiol. 80(4): 1860–1867. 10.1152/jn.1998.80.4.1860 9772245

[35] Hirayama M, Kawato M, Jordan MI (1993) The cascade neural network model and a speed-accuracy trade-off of arm movement. J Motor Behav. 25(3): 162–174. 10.1080/00222895.1993.9942047 12581987

[36] Scheidt RA, Ghez C (2007) Separate adaptive mechanisms for controlling trajectory and final position in reaching. J Neurophysiol. 98(6): 3600–3613 10.1152/jn.00121.2007 17913996

[37] Franklin DW, Milner TE (2003) Adaptive control of stiffness to stabilize hand position with large loads. Exp Brain Res. 152(2): 211–220 10.1007/s00221-003-1540-3. 10.1007/s00221-003-1540-3 12845511

[38] Franklin DW, Liaw G, Milner TE, Osu R, Burdet E, et al (2007) Endpoint stiffness of the arm is directionally tuned to instability in the environment. J Neurosci 27(29): 7705–7716 10.1523/JNEUROSCI.0968-07.2007 17634365 PMC6672883

[39] Sainburg RL (2005) Handedness: differential specializations for control of trajectory and position. Exerc Sport Sci Rev. 33(4): 206–213 10.1097/00003677-200510000-00010 PMC10709818 16239839

[40] Mutha PK, Sainburg RL (2007) Control of velocity and position in single joint movements. Hum Mov Sci. 26(6): 808–823 10.1016/j.humov.2007.06.001 PMC2607068 17931729

[41] Uno Y, Kawato M, Suzuki R (1989) Formation and control of optimal trajectory in human multijoint arm movement. Minimum torque-change model. Biol Cybern. 61(2): 89–101. 10.1007/BF00204593 2742921

[42] Harris CM, Wolpert DM (1998) Signal-dependent noise determines motor planning. Nature. 394(6695): 780–784. 10.1038/29528 9723616

[43] Berret B, Darlot C, Jean F, Pozzo T, Papaxanthis C, et al. (2008) The inactivation principle: mathematical solutions minimizing the absolute work and biological implications for the planning of arm movements. PLoS Comput Biol, 4 . doi: 10.1371/journal.pcbi.1000194. 10.1371/journal.pcbi.1000194 PMC2561290 18949023

[44] Nelson W (1983) Physical principles for economies of skilled movements. Biol Cybern. 46: 135–147 10.1007/BF00339982 6838914

[45] Todorov E, Jordan MI (2002) Optimal feedback control as a theory of motor coordination. Nat Neurosci. 5(11): 1226–1235 10.1038/nn963 12404008

[46] Shadmehr R, Arbib MA (1992) A mathematical analysis of the force-stiffness characteristics of muscles in control of a single joint system. Biol Cybern. 66(6): 463–477 10.1007/BF00204111 1586671

[47] Osu R, Gomi H (1999) Multijoint Muscle Regulation Mechanisms Examined by Measured Human Arm Stiffness and EMG Signals. J Neurophysiol. 81(4): 1458–1468. 10.1152/jn.1999.81.4.1458 10200182

[48] Gomi H, Osu R (1998) Task-dependent viscoelasticity of human multijoint arm and its spatial characteristics for interaction with environments. J Neurosci. 18(21): 8965–8978. 10.1523/JNEUROSCI.18-21-08965.1998 PMC6793558 9787002

[49] Mutha PK, Boulinguez P, Sainburg RL (2008) Visual mo dulation of proprioceptive reflexes during movement. Brain Res. 1246: 54–69 10.1016/j.brainres.2008.09.061 PMC2752307 18926800

[50] Scott S (2012) The computational and neural basis of voluntary motor control and planning. Trends in Cognitive Science. 16(11): 541–549 10.1016/j.tics.2012.09.008 23031541

[51] Franklin DW, Burdet E, OSU R, Kawato M, Milner TE (2003) Functional significance of stiffness in adaptation of multijoint arm movements to stable and unstable dynamics. Exp.Brain Res. 151(2): 145–157 10.1007/s00221-003-1443-3 12783150

[52] Takahashi CD, Scheidt RA, Reinkensmeyer DJ (2001) Impedance control and internal model formation when reaching in a randomly varying dynamical environment. J Neurophysiol. 86: 1047–1051. 10.1152/jn.2001.86.2.1047 11495973

[53] Wei K, Wert D, Körding K (2010) The nervous system uses nonspecific motor learning in response to random perturbations of varying nature. J Neurophysiol. 104: 3053–3063. 10.1152/jn.01025.2009 PMC3007651 20861427

[54] Sainburg RL, Kalakanis D (2000) Differences in control of limb dynamics during dominant and non-dominant arm reaching. J Neurophysiol. 83(5): 2661–2675. 10.1152/jn.2000.83.5.2661 PMC10709817 10805666

[55] Bagesteiro LB, Sainburg RL (2002) Handedness: dominant arm advantages in control of limb dynamics. J Neurophysiol. 88(5): 2408–2421. 10.1152/jn.00901.2001 PMC10709816 12424282

[56] Schaefer SY, Haaland KY, Sainburg RL (2007) Ipsilesional movement deficits in stroke: Accuracy deficits in right hemisphere patients are not due to faulty visuomotor transformations. Society of Neuroscience Abstracts.

[57] Schaefer SY, Haaland KY, Sainburg RL (2007) Hemispheric Specialization During Visually-Mediated Response Modifications. Motor Control 11 (Supplement): p. S113.

[58] Schaefer SY, Sainburg RL, Haaland KY (2005) Differential hemispheric contributions to unilateral arm movements. Society of Neuroscience Abstracts.

[59] Schaefer SY, Haaland KY, Sainburg RL (2009) Hemispheric specialization and functional impact of ipsilesional deficits in movement coordination and accuracy. Neuropsychologia. 47(13): 2953–2966 10.1016/j.neuropsychologia.2009.06.025 PMC2752301 19573544

[60] Schaefer SY, Haaland KY, Sainburg RL (2009) Dissociation of initial trajectory and final position errors during visuomotor adaptation following unilateral stroke. Brain Res. 1298: 78–91 10.1016/j.brainres.2009.08.063. 10.1016/j.brainres.2009.08.063 PMC3151492 19728993

[61] Mutha PK, Sainburg RL, Haaland KY (2011) Left parietal regions are critical for adaptive visuomotor control. J Neurosci. 31(19): 6972–6981 10.1523/JNEUROSCI.6432-10.2011 PMC3107546 21562259

[62] Mutha PK, Sainburg RL, Haaland KY (2011) Critical neural substrates for correcting unexpected trajectory errors and learning from them. Brain. 134(12): 3647–3661 10.1093/brain/awr275 PMC3235559 22075071

[63] Schaefer SY, Mutha PK, Haaland KY, Sainburg RL (2012) Hemispheric specialization for movement control produces dissociable differences in online corrections after stroke. Cereb Cortex. 22(6): 1407–1409 10.1093/cercor/bhr237 PMC3357180 21878488

[64] Mani S, Przybyla A, Good D, Haaland K, Sainburg RL (2013) Contralesional motor deficits after unilateral stroke reflect hemisphere-specific control mechanisms. Brain. doi: 10.1093/brain/aws283. 10.1093/brain/aws283 PMC3613707 23358602

